# Sex and diet, but not exercise, alter cardiovascular ACE2 and TMPRSS2 mRNA levels in aortic banded swine

**DOI:** 10.1152/japplphysiol.00736.2022

**Published:** 2023-01-19

**Authors:** Shannon C. Kelly, Pamela K. Thorne, Emily V. Leary, Craig A. Emter

**Affiliations:** ^1^Department of Biomedical Sciences, University of Missouri, Columbia, Missouri; ^2^NextGen Precision Health Institute, University of Missouri, Columbia, Missouri; ^3^Department of Orthopaedic Surgery, University of Missouri, Columbia, Missouri

**Keywords:** ACE2, exercise, heart failure, sex, TMPRSS2

## Abstract

SARS-COV-2, or COVID-19, is a respiratory virus that enters tissues via the angiotensin-converting enzyme 2 (ACE2) receptor and is primed and activated by transmembrane protease, serine 2 (TMPRSS2). An interesting dichotomy exists regarding the preventative/therapeutic effects of exercise on COVID-19 infection and severity. Although exercise training has been shown to increase ACE2 receptor levels (increasing susceptibility to COVID-19 infection), it also lowers cardiovascular risk factors, systemic inflammation, and preserves normal renin-angiotensin system axis equilibrium, which is considered to outweigh any enhanced risk of infection by decreasing disease severity. The goal of this study was to determine the effects of chronic exercise training, sex, and Western diet on ACE2 and TMPRSS2 mRNA levels in preclinical swine models of heart failure. We hypothesized chronic exercise training and male sex would increase ACE2 and TMPRSS2 mRNA levels. A retrospective analysis was conducted in previously completed studies including: *1*) sedentary and exercise-trained aortic banded male, intact Yucatan mini-swine (*n* = 6 or 7/group); *2*) ovariectomized and/or aortic banded female, intact Yucatan mini-swine (*n* = 5–8/group); and *3*) lean control or Western diet-fed aortic banded female, intact Ossabaw swine (*n* = 4 or 5/group). Left ventricle, right ventricle, and coronary vascular tissue were evaluated using qRT-PCR. A multivariable regression analysis was used to determine differences between exercise training, sex, and Western diet. Chronic exercise training did not alter ACE2 or TMPRSS2 level regardless of intensity. ACE2 mRNA was altered in a tissue-specific manner due to sex and Western diet. TMPRSS2 mRNA was altered in a tissue-dependent manner due to sex, Western diet, and pig species. These results highlight differences in ACE2 and TMPRSS2 mRNA regulation in an experimental setting of preclinical heart failure that may provide insight into the risk of cardiovascular complications of SARS-COV-2 infection.

**NEW & NOTEWORTHY** This retrospective analysis evaluated the impact of exercise, sex, and diet on ACE2 and TMPRSS2 mRNA levels in preclinical swine heart failure models. Unlike normal exercise intensities, exercise training of an intensity tolerable to a patient with heart failure had no influence on ACE2 or TMPRSS2 mRNA. In a tissue-specific manner, ACE2 mRNA levels were altered due to sex and Western diet, whereas TMPRSS2 mRNA levels were sensitive to sex, Western diet, and pig species.

## Graphical abstract

**Figure:**
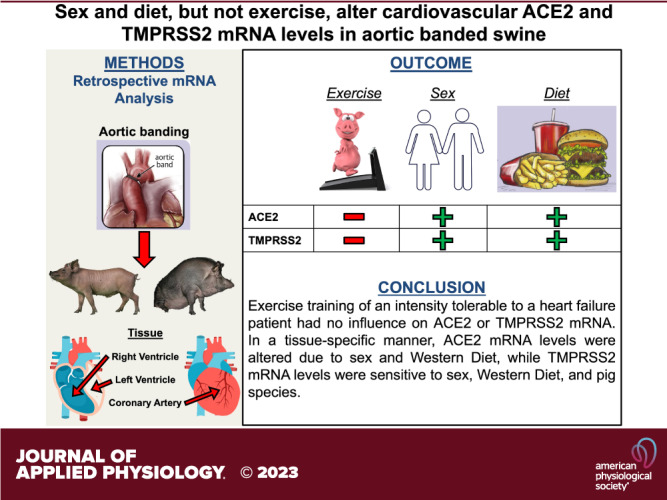

## INTRODUCTION

An interesting paradox exists regarding the preventative/therapeutic effects of exercise for acute respiratory syndrome coronavirus 2, or SARS-COV-2 (also known as COVID-19). SARS-COV-2 has numerous spike, or S, proteins on the viral envelope, which bind to the angiotensin-converting enzyme 2 (ACE2) receptor (1, 2). Exercise training has been shown to increase ACE2 receptor levels, potentially increasing susceptibility to COVID-19 infection (3). In contrast, exercise-dependent lowering of metabolic and cardiovascular risk factors, including decreased systemic inflammation by reducing ACE1:ACE2 ratios, is thought to outweigh any enhanced risk of infection (4). Binding of SARS-CoV-2 to ACE2 alone is not sufficient for cellular entry, as virus must also be primed at the plasma membrane by transmembrane protease, serine 2 (TMPRSS2; 1, 5). Although much attention has focused on ACE2 and TMPRSS2 levels in the lung, these proteins are also expressed in the heart and many other tissues (6–10). In comparison with ACE2, studies examining the impact of exercise on TMPRSS2 levels are extremely limited with a PubMed.gov search of exercise and TMPRSS2 returning only nine reports. An examination of these reports revealed five studies did not evaluate exercise and TMPRSS2, whereas the other four showed increased, decreased, or no change in TMPRSS2 mRNA or protein levels that were observed in a tissue/organ-dependent manner, demonstrating a lack of consistent findings in the currently available literature (11–14).

Elucidating the molecular mechanisms underlying exercise-dependent improvements to cardiovascular health in heart failure has been ongoing since exercise was recommended for this clinical population ∼20 years ago. Although studies indicate exercise generally increases ACE2 levels, it is unclear if exercise of an intensity tolerable to the patient with heart failure influences genetic regulation of either ACE2 or TMPRSS2. Heart failure is associated with more severe complications and higher mortality rates in individuals with SARS-COV-2 (15–17), and the virus itself has the potential to induce cardiac complications including heart failure (18–20). Risk for severe complications and increased mortality risk from SARS-CoV-2 is also linked to hypertension and metabolic disease (17, 21–23). Significant sex disparities from the virus have likewise been noted, with more severe complications associated with males despite similar infection rates between males and females (23–25).

The goal of this retrospective study was to determine ACE2 and TMPRSS2 mRNA levels in multiple swine models of pressure overload-induced heart failure to evaluate the effects of chronic exercise training tolerable to a patient with heart failure, sex, and Western diet on molecular mechanisms that may underlie observed connections between cardiovascular disease and SARS-COV-2 severity. Our laboratory has previously demonstrated that chronic cardiac pressure overload induced by aortic banding in swine, in the presence or absence of metabolic comorbidities induced by Western diet, generates relevant heart failure phenotypes (26–28). By examining left ventricle (LV), right ventricle (RV), and coronary tissue from these animals, a unique data set was developed providing rare insight into how ACE2 and TMPRSS2 gene expression is altered in preclinical swine models. Given available evidence demonstrating ACE2 levels are increased by exercise (3) and higher in men with heart failure (29), we hypothesized chronic exercise training and male sex would increase ACE2 and TMPRSS2 mRNA levels.

## METHODS

### Experimental Design

**Figure 1.**
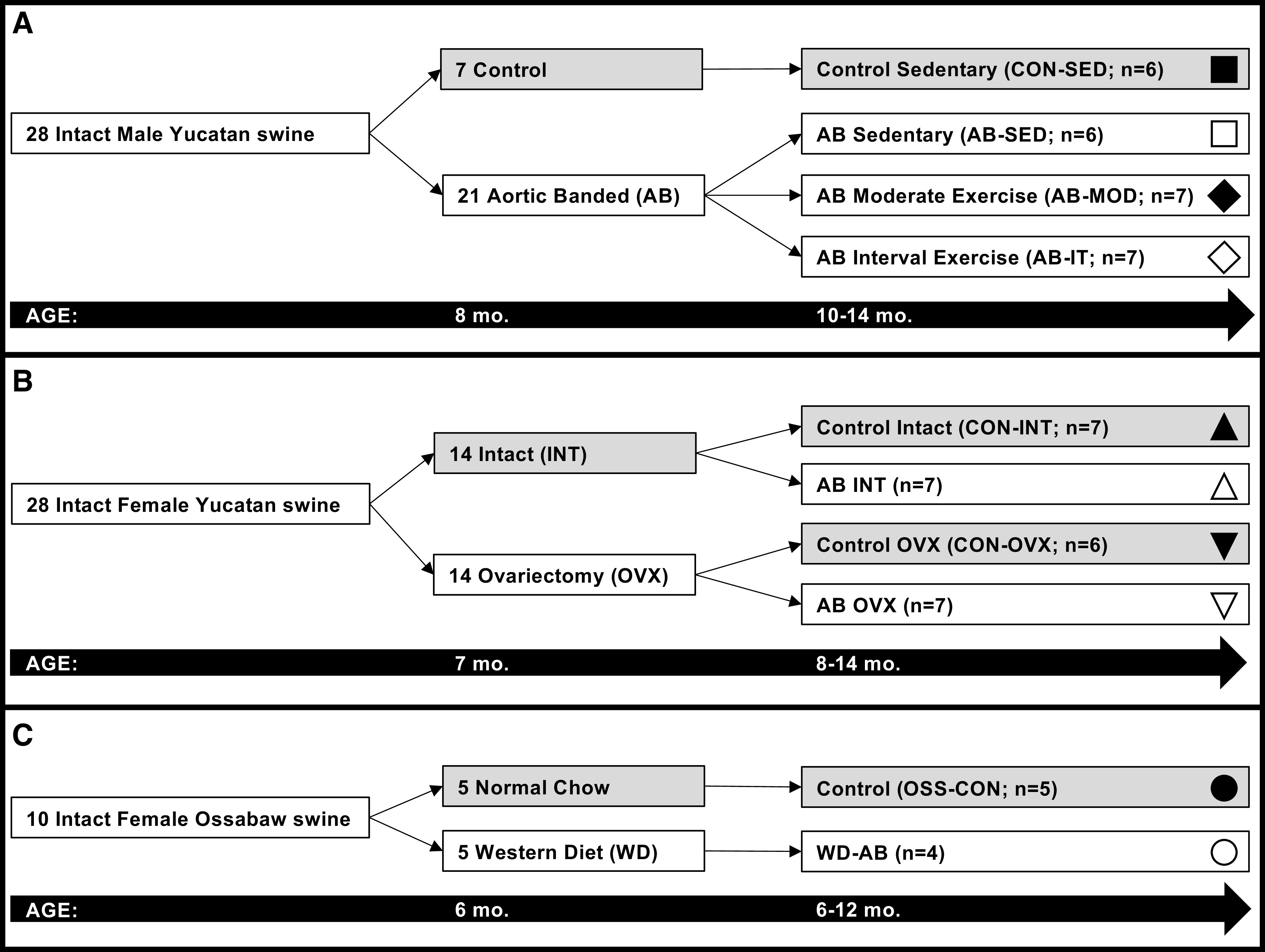
Schematic of experimental designs for the retrospective analysis of ACE2 and TMPRSS2 mRNA levels. Timeline of each study is shown below the study groupings. All animals were subject to 6 mo of pressure overload. Study design for *study 1* (*A*): Ref. 28; *study 2* (*B*): Ref. 27; *study 3* (*C*): Ref. 26. Key for Figs. 2 and 3 is provided: ▪ control sedentary (CON-SED); ≤ aortic banded sedentary (AB-SED); ♦ aoric banded moderate exercise trained (AB-MOD); ♢ aortic banded interval exercise trained (AB-IT); ▲ control intact (CON-INT); ▵ aortic banded intact (AB-INT); ▼ control ovariectomized (CON-OVX); ▿ aortic banded ovariectomized (AB-OVX); ● normal chow control (OSS-CON); ○ Western diet aortic banded (WD-AB). ACE2, angiotensin-converting enzyme 2; TMPRSS2, transmembrane protease, serine 2.

ACE2 and TMPRSS2 mRNA levels in LV, RV, and coronary arteries (resistance vessels or the right coronary artery) collected from 62 pigs (26 male and 36 female) were retrospectively analyzed. A graphical summary of the study designs for which these animals were used is presented in Fig. 1. In *study 1* (Fig. 1*A*; 28), 8-mo-old male intact Yucatan swine were assigned to sedentary control (CON-SED; *n* = 6) or chronic cardiac pressure overload-induced heart failure groups generated by aortic-banding (AB). Two months after surgery, AB animals were divided into three groups: sedentary (AB-SED; *n* = 6), interval-exercise trained (AB-IT; *n* = 7), or moderate-exercise trained (AB-MOD; *n* = 7). Exercise training consisted of treadmill running 55 min/day, 3 days/wk, for 17 wk with gradually increasing intensity using previously published protocols (28): *1*) interval exercise—*i*) a 5-min warm-up at 2 mph, *ii*) six 5-min sessions at 3 mph with five 3-min intervals at 4 mph in between, and *iii*) a 5-min cool down at 2 mph; and *2*) moderate exercise—*i*) a 5-min warm-up at 1.5 mph, *ii*) 45 min at 2.5 mph, and *iii*) a 5 min cool down at 1.5 mph. In *study 2* (Fig. 1*B*; 27), 7-mo-old female Yucatan swine were kept intact (INT) or ovariectomized (OVX). One month later, 50% of INT and OVX animals were further assigned to undergo aortic banding (AB) or remain a nonsurgical control (CON), resulting in four groups: CON-INT (*n* = 6); AB-INT (*n* = 7); CON-OVX (*n* = 6); or AB-OVX (*n* = 6). In *study 3* (Fig. 1*C*; 26), 2-mo-old female, intact Ossabaw swine were assigned to two groups: *1*) Western diet (WD) starting at 2 mo of age followed by aortic banding at 6 mo of age (WD-AB; *n* = 4); and *2*) normal diet nonsurgical control (OSS-CON; *n* = 5). The OSS-CON group was fed a standard chow diet (5L80, Lab Diet; 3.03 kcal·g^−1^, carbohydrate = 71%, protein = 18.5%, and fat = 10.5%; 500 g/day), whereas the WD-AB group was fed a Western diet (1,000 g/day) high in fat, high fructose corn syrup, and cholesterol [5B4L, Laboratory Diet; 4.14 kcal·g^−1^; carbohydrate, 40.8% (17.8% of total calories from high fructose corn syrup); protein, 16.2%; fat, 43%, 2% cholesterol wt/wt; 26]. Animals were fed once per day, and water was provided ad libitum. Hemodynamics for aortic banding procedures are shown in Table 1, and importantly demonstrate the physiologic insult used to stimulate the development of heart failure was equivalent across studies. All animal protocols for these studies were in accordance with the Principles for the Utilization and Care of Vertebrate Animals Used in Testing Research and Training and previously approved by the University of Missouri Animal Care and Use Committee (26–28).

**Table 1.** Aortic banding procedure hemodynamics

Group	Trans-Stenotic Systolic Pressure Gradient, mmHg	Peripheral Mean Arterial Pressure, mmHg	Heart Rate, beats/min
AB-SED	74 ± 5	90 ± 2	103 ± 14
AB-MOD	72 ± 3	89 ± 1	106 ± 14
AB-IT	74 ± 2	91 ± 2	99 ± 21
AB-INT	73 ± 8	90 ± 1	99 ± 14
AB-OVX	72 ± 8	89 ± 1	109 ± 12
WD-AB	72 ± 6	87 ± 6	84 ± 8

Values are means ± SD. No significant differences were found between groups. AB-INT, aortic banded intact; AB-IT, aortic banded interval exercise trained; AB-MOD, aortic banded moderate exercise trained; AB-OVX, aortic banded ovariectomized; AB-SED, aortic banded sedentary; WB-AB, Western diet aortic banded.

### qRT-PCR

Quantitative real-time PCR (qRT-PCR) was performed as previously described (26, 30). Target gene expression was normalized to 18S ribosomal RNA, and mRNA levels were measured using the following primers: 18S (F: 5′- CGGCTACCACATCCAAGGAA-3′; R: 5′- AGCTGGAATTACCGCGGC-3′), ACE2 (F: 5′- GGTAGTGGTCGGTATCGT-3′; R: 5′- TTTCTTCGCTGCTTGCTTGC-3′), and TMPRSS2 (F: 5′- AAGCGACTGGCTCCTCATTC-3′; R: 5′- AGGATCTCTGCGTTGACACG-3′).

### Statistical Analysis

A multivariable regression model, with physiologically important two-way interactions, was utilized to determine differences between six factors: pig species (Ossabaw or Yucatan), sex hormones (female intact, female ovariectomized, and male intact), aortic banding status (AB or non-AB), diet [Western diet (WD) or normal chow], exercise training (sedentary, moderate, and interval), and tissue type (LV, RV, and coronary). ACE2 and TMPRSS2 mRNA levels were log-transformed to meet linear regression assumptions. Data are presented as means ± standard deviation (SD). Pairwise comparisons were determined using least squares means. Significance is reported at the *P* < 0.10 and *P* < 0.05 levels (31, 32). Data analysis was performed with R version 4.2.1. (33), SAS 9.4TS Level 1M7, and Prism version 9.0.

## RESULTS

### ACE2 mRNA Levels

**Figure 2.**
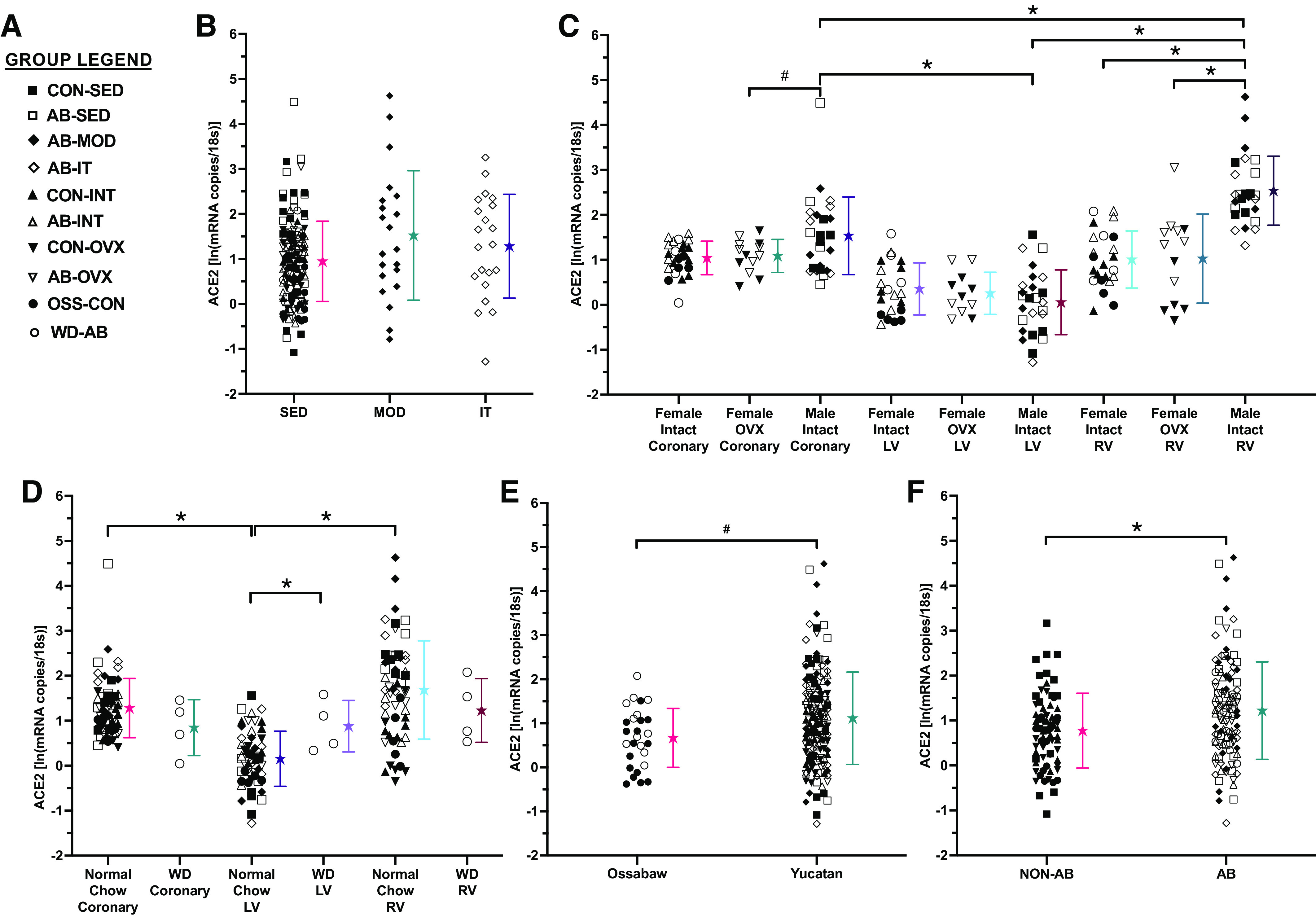
ACE2 mRNA levels are altered by sex, Western diet, tissue type, species, and aortic banding but not exercise*. A*: figure legend for experimental groups. *B*: chronic exercise training did not affect ACE2 mRNA level regardless of dose or intensity. Tissue-specific differences in ACE2 mRNA levels were dependent upon sex (*C*) and Western diet (*D*). ACE2 mRNA levels were significantly higher in Yucatan compared with Ossabaw swine (*E*) and following aortic banding compared with nonsurgical controls (*F*). ★ Mean ± SD for each group. #*P* < 0.10, *P < 0.05. AB, aortic banding; ACE2, angiotensin-converting enzyme 2; CON, control; INT, intact; IT, interval exercise trained; LV, left ventricle; MOD, moderate exercise trained; OSS, Ossabaw; OVX, ovariectomy; RV, right ventricle; SED, sedentary; WD, Western diet.

Multivariable regression analysis demonstrated ACE2 mRNA levels were dependent upon sex, tissue type, Western diet, pig species, and aortic banding (group legend presented in Fig. 2*A*). Chronic exercise training, regardless of dose or intensity, did not change ACE2 mRNA levels (Fig. 2*B*). However, significant differences in ACE2 mRNA levels for the LV, RV, and coronary vasculature were dependent upon sex independent of pig species, aortic banding, and exercise (tissue × sex hormones interaction; *P* < 0.001, Fig. 2*C*). Specifically, ACE2 mRNA levels were higher in male intact RV than male intact LV, male intact coronary vasculature, female intact RV, and female OVX RV. In addition, ACE2 mRNA levels in male intact coronary vasculature were higher than male intact LV and female OVX coronary vasculature. ACE2 mRNA levels per tissue type were dependent upon the sex of the animal rather than sex hormones present, as no differences were observed between intact and ovariectomized females.

Significant tissue differences in ACE2 mRNA levels were also dependent upon Western diet (tissue × Western diet interaction; *P* = 0.077, Fig. 2*D*), diet independent of aortic banding, sex, species, and exercise. Specifically, ACE2 mRNA levels were higher in the LV of animals fed a Western diet than animals eating normal chow. In addition, in animals fed normal chow, ACE2 mRNA levels in the RV and coronary vasculature were higher than the LV. ACE2 mRNA was also higher in Yucatan than Ossabaw swine (main effect of species; *P* = 0.095, Fig. 2*E*) and following aortic banding compared with nonsurgical controls (main effect of aortic banding; *P* = 0.02, Fig. 2*F*).

### TMPRSS2 mRNA Levels

**Figure 3.**
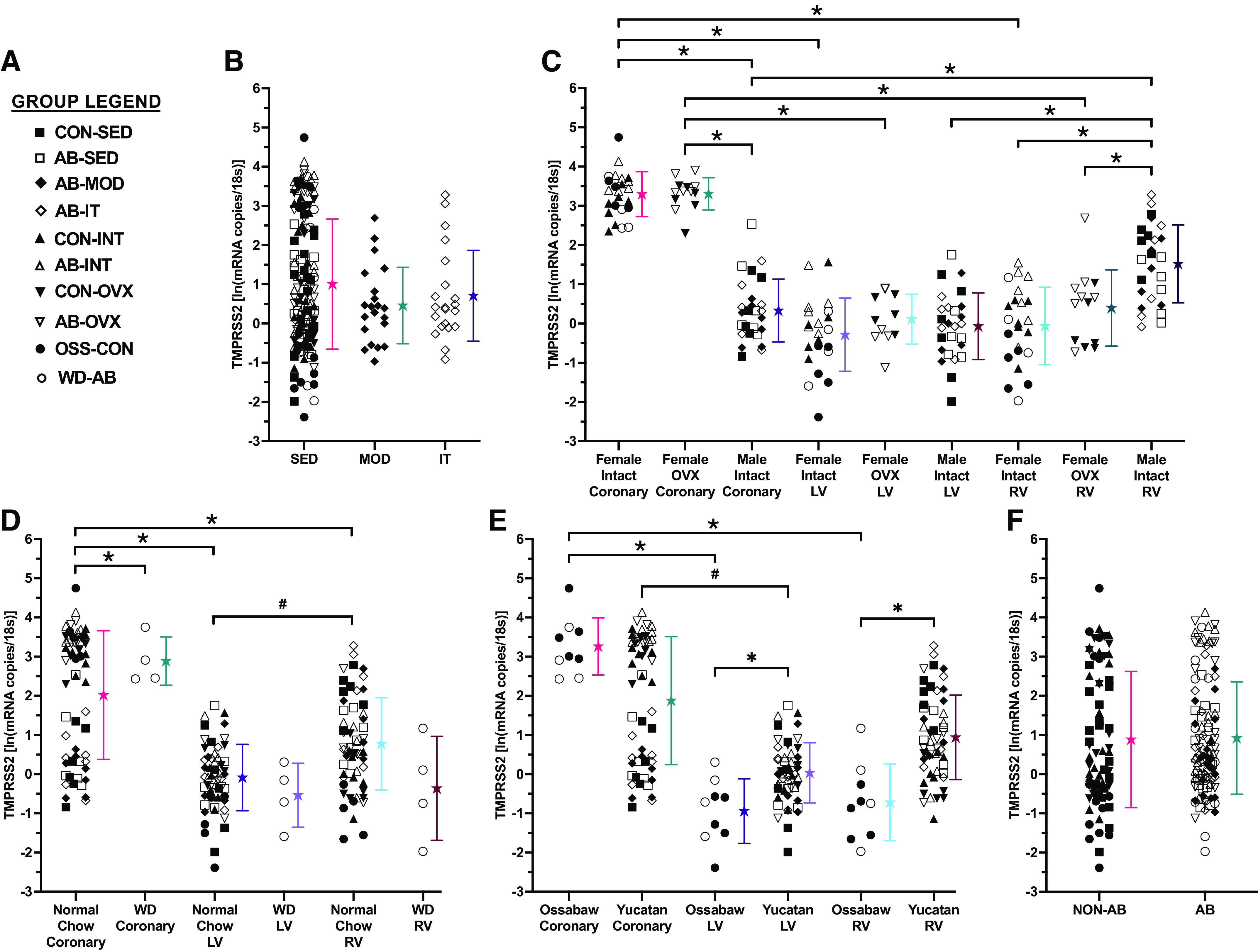
TMPRSS2 mRNA levels are altered by sex, Western diet, tissue type, and pig species but not exercise. *A*: figure legend for experimental groups. *B*: chronic exercise training did not affect TMPRSS2 mRNA level regardless of dose or intensity. Tissue specific differences in TMPRSS2 mRNA levels were dependent upon sex (*C*), Western diet (*D*), and pig species (*E*). TMPRSS2 mRNA levels were not changed by aortic banding (*F*). ★ Mean ± SD for each group. #*P* < 0.10, *P < 0.05. AB, aortic banding; CON, control; INT, intact; IT, interval exercise trained; LV, left ventricle; MOD, moderate exercise trained; OSS, Ossabaw; OVX, ovariectomy; RV, right ventricle; SED, sedentary; TMPRSS2, transmembrane protease, serine 2; WD, Western diet.

Differences in TMPRSS2 mRNA levels for the LV, RV, and coronary vasculature were separately dependent upon sex, Western diet, and pig species (group legend presented in Fig. 3*A*). TMPRSS2 mRNA levels were not altered due to chronic exercise training irrespective of dose or intensity (Fig. 3*B*). Similar to ACE2 mRNA, TMPRSS2 mRNA levels were higher in female intact coronary vasculature than female intact LV and RV tissue (tissue × sex hormones interaction; *P* < 0.001, Fig. 3*C*). Specifically, TMPRSS2 mRNA levels in female OVX coronary vasculature were higher than female OVX LV and RV tissue, and female intact and OVX coronary vasculature were also higher than male intact coronary vasculature. Male intact RV levels were higher than female intact and OVX RV levels as well as male intact LV and male intact coronary vasculature. Differences in TMPRSS2 mRNA levels were dependent upon the sex of the animal rather than sex hormones present given no differences were observed between female OVX and female intact tissues. These results were independent of aortic banding, Western diet, species, and exercise.

Tissue differences in TMPRSS2 mRNA levels were also dependent upon Western diet, independent of aortic banding, sex, species, and exercise (tissue × Western diet interaction; *P* = 0.08, Fig. 3*D*). Tissue differences were observed in animals fed normal chow as TMPRSS2 mRNA levels in these animals were higher in the coronary vasculature than LV and RV. In animals fed normal chow, TMPRSS2 mRNA was also higher in the RV than the LV. TMPRSS2 mRNA levels in the coronary vasculature of animals fed a Western diet were also higher than those fed normal chow.

In addition, tissue differences in TMPRSS2 mRNA levels were dependent upon pig species independent of aortic banding, sex, Western diet, and exercise (tissue × species interaction; *P* = 0.005, Fig. 3*E*). Higher mRNA levels were observed in the Yucatan LV than in Ossabaw LV, as well as in the Yucatan RV than in Ossabaw RV. The Ossabaw coronary vasculature was higher than the Ossabaw LV and RV, whereas Yucatan coronary vasculature was higher than the Yucatan LV. TMPRSS2 mRNA levels were not altered due to aortic banding (Fig. 3*F*).

## DISCUSSION

Contrary to our hypothesis, ACE2 and TMPRSS2 mRNA levels were not sensitive to a chronic exercise training stimulus tolerable to a patient with heart failure irrespective of total exercise dose or intensity. This is reflected by the increased intensity used in the interval compared with the moderate training protocol (3–4 mph vs. 2.5 mph, respectively), which also resulted in a greater total exercise dose given frequency (3 times/wk) and exercise time (55 min/session) was the same between exercise groups. Previous work has shown exercise increases skeletal muscle ACE2 in healthy young men (34) and the brains of spontaneously hypertensive rats (35) using higher intensities and frequencies than those used in our studies. More limited studies have demonstrated exercise in healthy mice does not change cardiac TMPRSS2 protein (14), although a cell culture model of exercise showed a “myokine cocktail” under simulated exercise conditions decreased TMPRSS2 mRNA level in bronchial epithelial cells (12). An interesting dichotomy has been proposed regarding the preventative/therapeutic effects of exercise on SARS-CoV-2 infection. Specifically, this paradox is characterized by: *1*) an increased risk of infection given exercise generally increases ACE2 levels; and *2*) a parallel reduction of disease severity following infection due to well-recognized reductions in cardiovascular risk and inflammation resulting from chronic exercise training (3). Interestingly, the results of this study suggest exercise tailored to the capabilities of a heart failure population may not influence the direct regulation of these genes. However, it is important to consider that physical activity could reduce the severity of SARS-CoV-2 infection given its positive influence on overall systemic inflammation and general cardiovascular risk (4).

ACE2 and TMPRSS2 mRNA levels did not generally increase in males, which also deviated from our hypothesis. Both males and females demonstrated cellular levels of these molecular targets could change in a tissue-dependent manner. ACE2 is a member of the renin-angiotensin-aldosterone system that counteracts the vasoconstrictive, proinflammatory, profibrotic, and prohypertrophic pathways of angiotensin II and is found on the X chromosome (36). The upregulation of ACE2 by estrogen, X chromosome inactivation, or decreased ACE2 methylation is hypothesized to offer an advantage to females over males by maintaining fundamental renin-angiotensin system-regulatory axis equilibrium after viral infection, including physiological ACE1:ACE2 ratios (37). However, the mechanism by which X chromosome-mediated regulation of ACE2 impacts SARS-CoV-2 infection and symptoms requires further investigation (25). Interestingly, ACE2 mRNA levels in different tissue types were dependent upon the sex of the animal rather than the presence of sex hormones, as no differences between intact and ovariectomized females were observed. These findings were surprising, given sex hormones like estrogen are known to impact the regulation of ACE2 and suggest cellular control via the chromosome may be more influential. Previous studies have shown that soluble ACE2 plasma protein levels are increased in males compared with females in individuals with heart failure (29); however, ACE2 mRNA levels in the heart and blood vessels are increased in Asian females compared with Asian males (38). The current analysis supports the idea that tissue-specific differences based on sex could impact susceptibility to infection and disease severity in an experimental setting of heart failure, as ACE2 and TMPRSS2 levels differed between ventricular and coronary tissues in male and female animals.

In swine subject to aortic banding-induced heart failure, ACE2 mRNA was higher overall. Previous studies have found similar increases in ACE2 expression through RNA sequencing and/or proteomics in the heart of individuals with heart failure and aortic stenosis, as well as in mice that have undergone transaortic constriction (8, 39). Considered together, these data provide further support for the idea that individuals with heart failure may be more susceptible to severe complications from SARS-CoV-2 due to increased ACE2 levels.

The current report shows ACE2 mRNA levels were higher in the LV of swine fed a Western diet than animals fed normal chow. Interestingly, tissue-specific differences in the regulation of ACE2 mRNA in animals fed a normal chow diet were largely lost with a Western diet. It is possible that in healthy individuals, differential expression of ACE2 per tissue/organ system is essential to normal physiologic balance of the renin-angiotensin system and that loss of this heterogeneity negatively influences susceptibility and severity of a COVID-19 infection. The general loss of tissue-dependent differences in both ACE2 and TMPRSS2 mRNA levels in animals fed a Western diet in the current study coincides with clinical observations linking obesity with COVID-19 infection and severity (4). This idea further extends to individuals with heart failure, who may be predisposed to severe complications from COVID-19 due to comorbidities including obesity and diabetes (15). ACE2 expression was not altered in the heart due to high-fat diet in mice (9), suggesting species-dependent differences in the response of this gene to diet. Collectively, these findings imply tissue-specific differences based on a diet may alter susceptibility to SARS-CoV-2 infection and the risk of severe complications.

This novel preclinical investigation demonstrates TMPRSS2 mRNA levels are dependent upon sex, tissue type, Western diet, and pig species. TMPRSS2 is a protease whose physiological function and regulation are unknown (40), with limited to nonexistent examinations of the impact of sex or metabolic syndrome on its regulation. Its involvement in pathology includes priming viruses for cellular entry and upregulation in prostate cancer (41), and previous studies have shown that TMPRSS2’s transcription can be stimulated by androgens in the prostate but not in the lung (41, 42). These findings suggest regulation of TMPRSS2 by androgens may underlie tissue-specific differences based on sex. Increased TMPRSS2 expression in the LV and RV of Yucatan compared with Ossabaw swine imply genetics may also play a role in TMPRSS2 mRNA levels. This translational retrospective provides novel insight into sex hormone-mediated control of TMPRSS2 in the heart and coronary vasculature in a context of existing metabolic syndrome and cardiovascular disease, suggesting further scrutiny of sex and gene-based differences is warranted.

### Conclusions

This retrospective analysis presented a unique opportunity to evaluate the influence of exercise, sex, and diet on ACE2 and TMPRSS2 mRNA levels in a setting of experimental heart failure. Surprisingly, ACE2 or TMPRSS2 mRNA was not sensitive to exercise, suggesting training intensities tolerable for a patient with heart failure may not be potent enough to induce alterations in the regulation of these genes. In a tissue-specific manner, ACE2 mRNA levels were altered by sex and Western diet with TMPRSS2 mRNA levels influenced by sex, Western diet, and pig species. The findings highlight significant differences from preclinical models with relevant heart failure phenotypes, providing insight regarding increased complications from COVID-19 infection.

## DATA AVAILABILITY

Data will be made available upon reasonable request.

## GRANTS

This study was supported by a NIH R01 HL112998 (PI: C. A. Emter).

## DISCLOSURES

No conflicts of interest, financial or otherwise, are declared by the authors.

Craig Emter is an editor of *Journal of Applied Physiology* and was not involved and did not have access to information regarding the peer-review process or final disposition of this article. An alternate editor oversaw the peer-review and decision-making process for this article.

## AUTHOR CONTRIBUTIONS

S.C.K. and C.A.E. conceived and designed research; S.C.K., P.K.T., and C.A.E. performed experiments; S.C.K., P.K.T., E.V.L., and C.A.E. analyzed data; S.C.K., P.K.T., E.V.L., and C.A.E. interpreted results of experiments; S.C.K., E.V.L., and C.A.E. prepared figures; S.C.K., E.V.L., and C.A.E. drafted manuscript; S.C.K., P.K.T., E.V.L., and C.A.E. edited and revised manuscript; S.C.K., P.K.T., E.V.L., and C.A.E. approved final version of manuscript.
